# Inhibition of Vascular Endothelial Growth Factor Protects against the Development of Oxaliplatin-Induced Sinusoidal Obstruction Syndrome in Wild-Type but Not in CD39-Null Mice

**DOI:** 10.3390/cancers14235992

**Published:** 2022-12-05

**Authors:** Sebastian Knitter, Gregor Duwe, Anika Sophie Beierle, Sina Pesthy, Paul Viktor Ritschl, Karl Herbert Hillebrandt, Alexander Arnold, Thomas Malinka, Dominik Paul Modest, Marcus Bahra, Johann Pratschke, Igor Maximilian Sauer, Moritz Schmelzle, Andreas Andreou

**Affiliations:** 1 Charité-Universitätsmedizin Berlin, Corporate Member of Freie Universität Berlin and Humboldt-Universität zu Berlin, Department of Surgery, Campus Charité Mitte and Campus Virchow-Klinikum, Experimental Surgery, 10117 Berlin, Germany; 2 Berlin Institute of Health (BIH), 10178 Berlin, Germany; 3 Institute of Pathology, Charité-Universitätsmedizin Berlin, Corporate Member of Freie Universität Berlin and Humboldt-Universität zu Berlin, 10117 Berlin, Germany; 4 Charité-Universitätsmedizin Berlin, Corporate Member of Freie Universität Berlin and Humboldt-Universität zu Berlin, Department of Hematology, Oncology, and Cancer Immunology (CCM/CVK), 10117 Berlin, Germany; 5 Berlin School of Integrative Oncology, 13353 Berlin, Germany

**Keywords:** colorectal liver metastases, sinusoidal obstruction syndrome, bevacizumab

## Abstract

**Simple Summary:**

For patients undergoing multimodal treatment for colorectal liver metastases (CLM), the development of sinusoidal obstruction syndrome (SOS) as a side effect of oxaliplatin-based chemotherapy may endanger the prospects after liver resection. In this study, we aimed to investigate possible protective effects of an additional preoperative inhibition of *vascular endothelial growth factor* (VEGF) on the occurrence of SOS and its implications on liver function and regeneration after liver resection. After successful establishment of a novel murine model of SOS, we were able to show a reduced incidence of SOS after additive treatment with a VEGF inhibitor. Changes in the VEGF pathway, namely in the expression of *VEGF receptor-2* (VEGF-R2), may be responsible for these findings. By preventing the incidence of SOS, the inhibition of VEGF may help to reduce morbidity after liver resection for patients with CLM. Further clinical studies are needed to corroborate our results.

**Abstract:**

(1) *Background*: Sinusoidal obstruction syndrome (SOS) after oxaliplatin-based chemotherapy is associated with unfavorable outcomes after partial hepatectomy for colorectal liver metastases (CLM). Bevacizumab, a monoclonal antibody against *vascular endothelial growth factor* (VEGF), may prevent SOS development. We investigated the impact of VEGF-inhibition on the development of SOS in a murine model. (2) *Methods*: Male wild-type and CD39-null mice received oxaliplatin, additional anti-VEGF (OxAV), or controls, and were sacrificed or subjected to major partial hepatectomy (MH). Specimen were used for histological analysis of SOS. Liver damage was assessed by plasma transaminases. The VEGF pathway was elucidated by quantitative PCR of liver tissue and protein analysis of plasma. (3) *Results*: Mice treated with oxaliplatin developed SOS. Concomitant anti-VEGF facilitated a reduced incidence of SOS, but not in CD39-null mice. SOS was associated with increased plasma VEGF-A and decreased *hepatocyte growth factor* (HGF). After OxAV treatment, VEGF-R2 was upregulated in wild-type but downregulated in CD39-null mice. Oxaliplatin alone was associated with higher liver damage after MH than in mice with concomitant VEGF-inhibition. (4) *Conclusions*: We established a murine model of oxaliplatin-induced SOS and provided novel evidence on the protective effect of VEGF-inhibition against the development of SOS that may be associated with changes in the pathway of VEGF and its receptor VEGF-R2.

## 1. Introduction

As a part of multimodal treatment concepts for patients with colorectal liver metastases (CLM) [1], the neoadjuvant use of oxaliplatin-based chemotherapy before liver surgery is associated with the development of a specific liver injury known as sinusoidal obstruction syndrome (SOS) [2,3,4]. Oxaliplatin-induced SOS is a histologically defined illness that may affect up to 60% of patients undergoing partial hepatectomy for CLM [2,5,6], and is reportedly associated with increased perioperative morbidity including an increased risk for intraoperative blood loss with need for perioperative transfusions [5,7], higher liver failure rates [6,8], and early tumor recurrence and diminished long-term survival rates [9,10]. VEGF was found to be elevated in liver tissue and plasma of patients with SOS [11,12], which may provide a good hypothesis that the addition of bevacizumab (an inhibitor of *vascular endothelial growth factor*, VEGF) to oxaliplatin-based neoadjuvant regimens is associated with protective effects against the development of SOS [13]. The pathophysiological background leading to the development of SOS as well as the mechanisms of action of the inhibition of VEGF in suppressing SOS remain unclear.

CD39 (*cluster of differentiation 39)*—also known as ectonucleoside triphosphate diphosphohydrolase-1/ENTPD1—is a ubiquitous occurring, cell-surface located enzyme that is involved in purinergic signaling by catalyzing the hydrolysis of triphospho- and diphosphonucleosides to monophosphonucleoside derivatives and phosphate [14,15,16]. In the liver, CD39 plays an essential role in the response to acute toxic liver injury and promotion of liver regeneration [17]. In mice that do not express CD39, the communication between VEGF and its VEGF-receptor 2 (VEGF-R2) is impaired, leading to a diminished hepatic regenerative capacity [18,19].

The aims of the current study were (1) to establish a murine model of SOS induced by oxaliplatin alone, (2) to investigate the protective effects of VEGF-inhibition in wild type and CD39-null mice, and (3) to analyze the impact of SOS on liver regeneration and liver damage after partial hepatectomy.

## 2. Materials and Methods

### 2.1. In-Vivo Model of Oxaliplatin-Induced Sinusoidal Obstruction Syndrome

**Figure 1:**
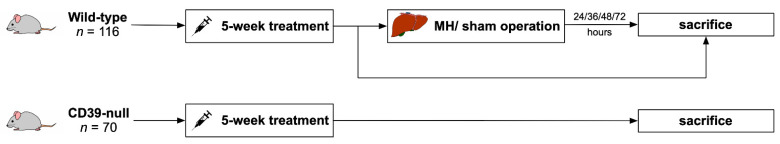
Project overview. wt (*n* = 116) and CD39-null mice (*n* = 70) received weekly intraperitoneal injections of their respective treatment (oxaliplatin alone, oxaliplatin and anti-VEGF, anti-VEGF alone, or glucose) over a time period of five weeks. Afterwards, wt mice were subjected to MH or sacrifice based on their general health. Instead of MH, some received a sham operation. Mice were sacrificed after 24, 36, 48, or 72 h after MH. All CD39-null mice were sacrificed after the five-week treatment period.

All animal experiments were approved by the local animal welfare authorities (*Landesamt für Gesundheit und Soziales* [LAGeSo], reference number: G 0053/16). Five- to eleven-week-old, male wild-type (wt, C57Bl6/N; *n* = 116) and CD39-null mice (cd39, *n* = 70) were randomly assigned different treatment: oxaliplatin alone (wtOx, *n* = 40; cd39Ox, *n* = 24), oxaliplatin and anti-VEGF (wtOxAV, *n* = 40; cd39OxAV, *n* = 12), anti-VEGF alone (wtAV, *n* = 12; cd39AV, *n* = 12), and glucose (wtGlu, *n* = 24; cd39Glu, *n* = 22). After completion of a five-week treatment period, animals were either sacrificed or subjected to major partial hepatectomy (MH) with removal of 70% of liver mass (see Appendix A and Figure 1). After MH, mice were culled after randomly allocated time points (24, 36, 48, or 72 h).

### 2.2. Processing of Liver Specimen: Histological Assessment of SOS, Liver Regeneration and Quantitative PCR

Liver specimens were analyzed for histological evidence of SOS adapted to the definition of Rubbia-Brandt et al. [2] (see Appendix B). Evaluation of slides was performed blinded to treatment and control groups. Parameters included presence and grading of sinusoidal dilation (grade 0 to 3), presence of perisinusoidal hemorrhage, grading of nodularity (grade 0 to 3), presence and grading of steatosis (grade 0 to 3), presence of steatohepatitis, and presence of hepatocellular damage. Liver regeneration was assessed by estimating liver mass increase by body and liver weight and immunohistological staining for Ki-67 and *bromodeoxyuridine* (BrdU). Liver tissue was also used for quantitative PCR for VEGF-A, VEGF-R1, and VEGF-R2 (see Appendix B).

### 2.3. Plasma Analysis of Parameters of Liver Damage and Other Analytes

Blood plasma obtained at sacrifice was analyzed for parameters of liver damage and function (aspartate transaminase, AST, alanine transaminase, ALT, bilirubin, and albumin), and factors which are part of the VEGF pathway (*hepatocyte growth factor*, HGF, *matrix metalloproteinase-9*, MMP-9, and *tissue inhibitor of metalloproteinases-1*, TIMP-1; Appendix B).

### 2.4. Statistical Analysis

Quantitative and qualitative variables were expressed as medians (range) and frequencies. The Chi-square or Fisher’s exact test, and the Mann–Whitney *U* test were used to compare categorical and continuous variables, as appropriate. When comparing more than two groups, one-way ANOVA with Tukey post-hoc method was used for parametric data. In case of non-parametric data, Kruskal–Wallis H test followed by Dunn post-hoc test with Bonferroni correction for multiple testing was conducted. When comparing two independent variables, two-way ANOVA with Tukey post-hoc tests were performed. *p* values ≤ 0.05 were considered statistically significant. SPSS software package, version 25, by IBM (Armonk, NY, USA) was used.

## 3. Results

### 3.1. Establishment of a New Murine Model of Oxaliplatin-Induced SOS and the Effect of VEGF-Inhibition on the Development of SOS

**Figure 2:**
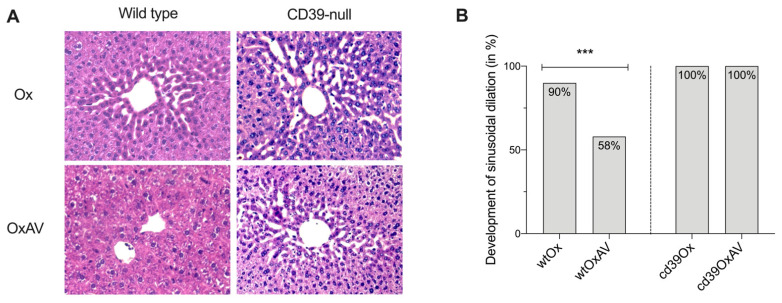
Histopathological analysis. (**A**) Characteristic H&E slides of liver tissue of wt and CD39-null mice after treatment with oxaliplatin or oxaliplatin and anti-VEGF: The typical sinusoidal dilation around the central veins is visible in all groups except wtOxAV. Magnification 20×. (**B**) Development of sinusoidal dilation as evaluated by H&E slides: Sinusoidal dilation was found in 90% and 58% in the wtOx and wtOxAV group, respectively (***, *p* = 0.001). In contrast, all CD39-null mice treated with oxaliplatin or oxaliplatin and anti-VEGF developed sinusoidal dilation. Hence, anti-VEGF did not protect against the development of sinusoidal dilation in CD39-null mice receiving oxaliplatin compared to wtOxAV (100% vs. 58%, *p* = 0.005).

In total, 116 wt mice and 70 CD39-null mice were used in this study. Blinded histopathological analysis of H&E-stained sections showed the development of sinusoidal dilation in 90% and 58% of wt mice treated with Ox and OxAV (*p* = 0.001, Figure 2), respectively, whereas sinusoidal dilation was absent in all animals of the wt control groups (*p* < 0.0001; Table 1). In 70 CD39-null mice, sinusoidal dilation was found in 100%, 100%, 0%, and 0% in mice treated with oxaliplatin (cd39Ox), oxaliplatin and anti-VEGF (cd39OxAV), anti-VEGF (cd39AV), and glucose (cd39Glu), respectively (*p* < 0.0001; Table 1). In subgroup analyses of mice receiving Ox, the presence of sinusoidal dilation showed no significant differences between wt and CD39-null mice (90% vs. 100%, *p* = 0.288). However, grading of sinusoidal dilation was more advanced in cd39Ox mice (*p* = 0.002). Perisinusoidal hemorrhage (*p* = 0.702), steatosis (*p* = 0.051), and hepatocellular damage (*p* = 0.372) were equivalent between wtOx and cd39Ox. Comparing OxAV mice, wt mice developed less frequent (58% vs. 100%, *p* = 0.005) and, in terms of grading, less advanced (*p* = 0.006) sinusoidal dilation compared to CD39-null mice (Table 1).

### 3.2. Pathogenesis of SOS: Quantitative PCR of Liver Tissue and Blood Plasma Analysis

**Figure 3:**
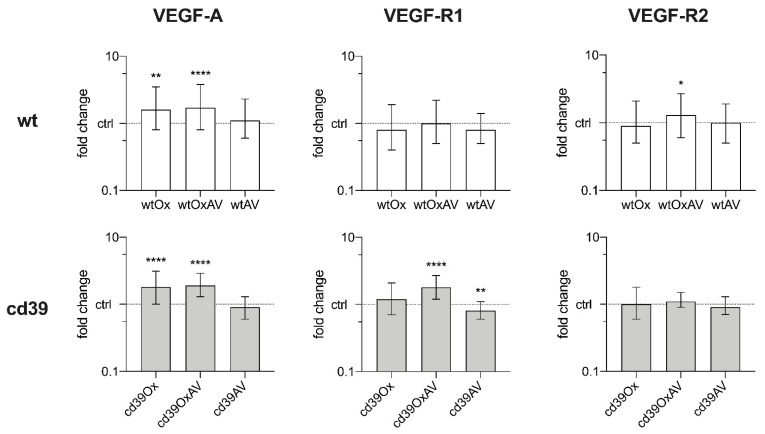
Analysis of quantitative PCR data in resected liver specimen. Median fold change and standard error. wtGlu and cd39Glu served as controls. (* *p* ≤ 0.05, ** *p* ≤ 0.01, **** *p* ≤ 0.0001) (**Left column**). VEGF-A. Expression of VEGF-A was significantly up-regulated in mice receiving oxaliplatin-containing regimens (wtOx: *p* = 0.004, wtOxAV: *p* < 0.0001, cd39Ox: *p* < 0.0001, and cd39OxAV: *p* < 0.0001). However, VEGF-A was equivalently expressed between groups receiving oxaliplatin (wtOx/wtOxAV: *p* = 0.786, cd39Ox/cd39OxAV: *p* = 0.990). (**Middle column**). VEGF-R1. In wt mice, VEGF-R1 expressions were comparable. In cd39-null mice compared to controls, VEGF-1 was equivalently expressed in cd39Ox (*p* = 0.137), up-regulated in cd39OxAV (*p* < 0.0001), and down-regulated in cd39AV (*p* = 0.007). (**Right column**). VEGF-R2. Most importantly, VEGF-R2 was up-regulated in wtOxAV (*p* = 0.045) compared to controls, while it was comparable among cd39-null mice (*p* = 0.112).

Specimen were further examined in quantitative PCR analysis (Figure 3 and Appendix C, Table A1): Among wt mice, VEGF-A was significantly up-regulated in wtOx and wtOxAV compared to controls, while being comparable between these two groups. VEGF-R1 expressions did not show any differences. Importantly, VEGF-R2 was up-regulated in wtOxAV. The analysis of CD39-null mice revealed the up-regulation of VEGF-A in cd39Ox and cd39OxAV compared to controls, whereas no significant differences were found between these two groups. VEGF-R1 was significantly upregulated in cd39OxAV and downregulated in cd39AV, but equivalently expressed in cd39Ox. VEGF-R2 was also equivalently expressed in all CD39-null mice.

**Figure 4:**
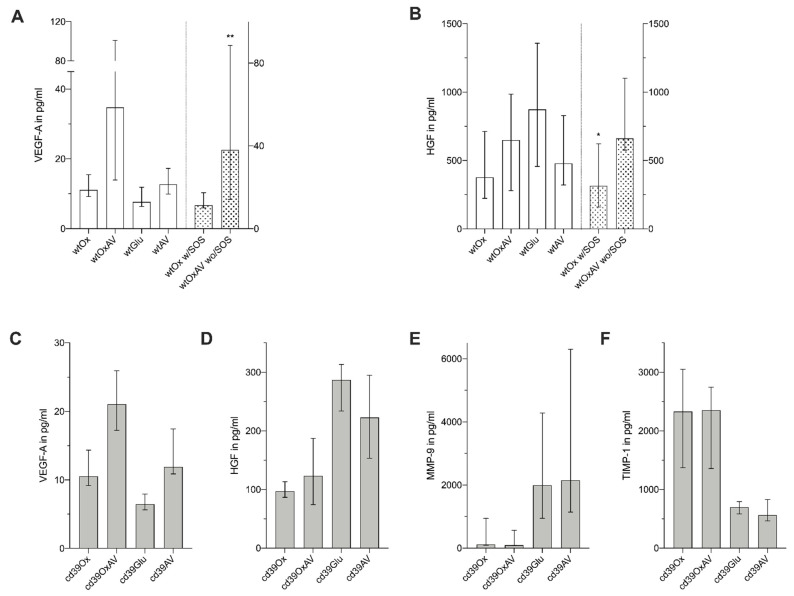
Results of magnetic bead-based assay of blood plasma. Medians and interquartile range. (* *p* ≤ 0.05, ** *p* ≤ 0.01) (**A**,**B**) VEGF-A (*p* < 0.0001, **A**) and HGF (*p* = 0.030, **B**) were significantly different among wt mice. The highest values for VEGF-A were found in the wtOxAV group (**A**). For HGF, lowest values were found in the wtOx group (**B**). In the subgroup comparison of wtOx mice with sinusoidal changes and wtOxAV mice without any histological changes, VEGF-A (*p* = 0.003, **A**) and HGF (*p* = 0.040, **B**) were significantly increased for wtOxAV. (**C**) cd39OxAV showed the highest values for VEGF-A; however, it was equivalent to cd39AV (*p* = 0.193). (**D**) Lowest values for HGF among cd39-null mice were measured for cd39Ox, while control mice (cd39Glu) showed significantly higher values (*p* < 0.0001). (**E**,**F**) cd39-null mice receiving oxaliplatin (cd39Ox and cd39OxAV) showed the lowest values for MMP-9 (**E**) and the highest values for TIMP-1 (**F**).

Possible factors associated with the development of sinusoidal dilation in the plasma of the mice were elucidated by a magnetic bead-based multiplex assay (Figure 4 and Appendix D). At time of sacrifice, significant differences between wt groups were revealed for VEGF-A (Figure 4A) and HGF (Figure 4B). Equivalent results were found for MMP-9 and TIMP-1. wtOxAV had the highest value of VEGF-A that was significantly different to wtOx and wtGlu, but not to wtAV (Figure 4A). Post-hoc analysis of HGF showed that it was lowest in wtOx (Figure 4B). Subgroup analysis of wild-type mice who developed SOS after Ox treatment and those that did not develop SOS after additional VEGF-inhibition revealed significant differences for VEGF-A and HGF (Figure 4A,B). All analyzed parameters were shown to be significantly different in the comparison of CD39-null mice (Figure 4C–F): Highest values of VEGF-A were measured in cd39OxAV that were significantly higher than cd39Ox and cd39Glu, but equivalent to cd39AV (Figure 4C). cd39Ox mice showed the lowest values of HGF (Figure 4D). Interestingly, MMP-9 was lowest in both cd39Ox and cd39OxAV (Figure 4E), whereas TIMP-1 was highest in cd39Ox (Figure 4F). Comparing cd39Ox and cd39OxAV, equivalent results were found for HGF, MMP-9, and TIMP-1 (Figure 4C–F).

### 3.3. Impact of SOS on Liver Damage and Liver Regeneration after Major Partial Hepatectomy

**Figure 5:**
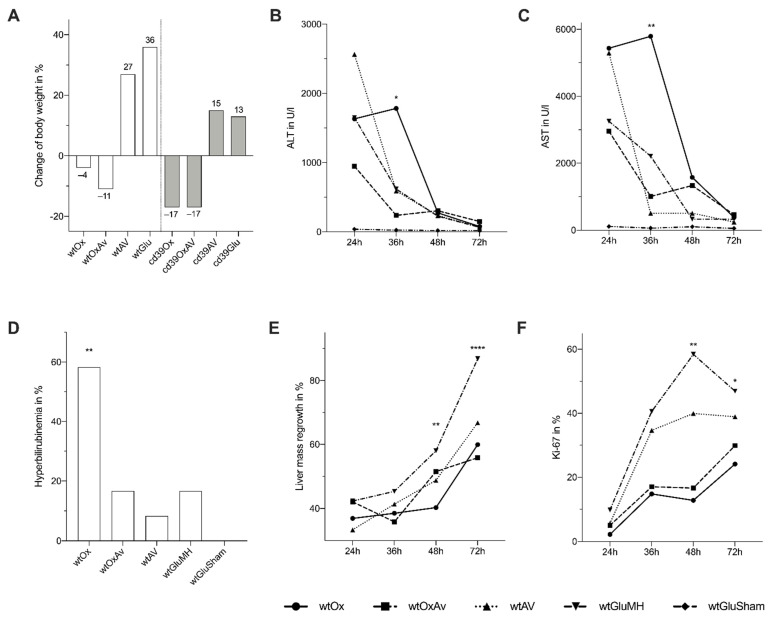
Course of treatment and results after major partial hepatectomy. (* *p* ≤ 0.05, ** *p* ≤ 0.01, **** *p* ≤ 0.0001) (**A**) Relative change of body weight of animals during the course of treatment (day of first treatment until MH or sacrifice) was −4%, −11%, +27%, and +36% in wtOx, wtOxAV, wtAV, and wtGlu, respectively (*p* < 0.0001), and −17%, −17%, +15%, and +13% in cd39Ox, cd39OxAV, cd39AV, and cd39Glu, respectively (*p* < 0.0001). Hence, loss of body weight was observed in all animals receiving systemic chemotherapy. (**B**,**C**) Plasma was obtained to compare liver parameters. Statistically significant interaction between intervention group and time after MH was found for ALT (*p* = 0.003; **B**) and AST (*p* = 0.002; **C**). Highest values for ALT and AST at 36 h after MH were found for wtOx (ALT: *p* = 0.035, AST: *p* = 0.004). (**D**) Hyperbilirubinemia after MH was observed in 58%, 17%, 8%, 17%, 0% in wtOx, wtOxAV, wtAV, wtGluMH, and wtGluSham, respectively (*p* = 0.004). (**E**) Liver mass regrowth after MH (*p* < 0.0001). Mice treated with oxaliplatin showed significantly reduced liver regrowth in comparison to wtGlu at 48 (*p* = 0.003) and 72 h (*p* < 0.0001). wtOxAV did not promote improved liver regrowth after MH. (**F**) Immunohistochemistry for Ki-67. Indices for wtOx and wtOxAV were equivalent at all times after MH. However, Ki-67 indices were reduced for wtOx after 36, 48, and 72 h after MH in comparison to wtAV (*p* = 0.089, *p* = 0.004 and *p* = 0.021, respectively) and wtGlu (*p* = 0.002, *p* < 0.0001 and *p* = 0.012, respectively). In contrast, Ki-67 indices of wtOxAV were only inferior compared to wtGlu at 36 (*p* = 0.008) and 48 h (*p* < 0.0001).

Mice who received systemic chemotherapy lost a significant amount of body weight during treatment (*p* < 0.0001; Figure 5A). Parameters of liver damage (AST, ALT) gradually declined over time after MH until they were comparable between all groups including sham-operated mice at 48 and 72 h (Figure 5B,C). At 36 h, the highest levels of AST and ALT were measured in wtOx, which were significantly different to wtOxAV (AST: *p* = 0.004, ALT: *p* = 0.035) and controls. Hyperbilirubinemia after MH was most frequently present in wtOx (58%, *p* = 0.004; Figure 5D).

Liver regeneration was assessed by weighing the liver specimen and calculating the relative regenerated liver weight increase of original liver weight (Figure 5E). wtOx showed significantly reduced liver regrowth in comparison to wtGlu at 48 (*p* = 0.003) and at 72 h (*p* < 0.0001). However, additional VEGF-inhibition was not able to facilitate improved liver regeneration based on liver regrowth as it was equivalent to wtOx at all time points after MH. Liver regeneration was further analyzed by immunohistochemistry for BrdU and Ki-67 (Figure 5F). A significant interaction effect between treatment and time after MH was found for Ki-67 (*p* = 0.002; Figure 5F) but not for BrdU (*p* = 0.135; data not shown). wtOx showed reduced indices for Ki-67 at 36, 48, and 72 h after MH in comparison to wtAV and wtGlu (Figure 5F). However, no significant differences could be found between wtOx and wtOxAV.

In our preliminary experiments, survival after MH for CD39-null mice who received oxaliplatin (cd39Ox and cd39OxAV) was significantly reduced. Therefore, we decided not to further perform any MH for CD39-null mice in order to comply with local animal welfare law and to ensure animal safety. CD39-null mice were sacrificed one week after last treatment without performing MH. Plasma analysis of liver parameters at sacrifice showed no significant differences between all groups for AST (*p* = 0.805), ALT (*p* = 0.539), and albumin (*p* = 0.163).

## 4. Discussion

In this study, we established a murine model of oxaliplatin-induced SOS in wt and CD39-null mice. After a five-week treatment period using oxaliplatin, analysis of resected liver specimen revealed histologically evident signs of sinusoidal dilation in 90% and 100% of wt and CD39-null mice, respectively. Concomitant administration of oxaliplatin and a murine VEGF-antibody was able to facilitate a significant reduction of frequency and severity of sinusoidal dilation in wt mice compared to treatment with oxaliplatin alone. However, the protective effect of additional VEGF-inhibition was absent in CD39-null mice compared to wt mice.

Previous animal models of SOS have mostly used monocrotaline (MCT), a pyrrolizidine alkaloid of plant origin, in order to induce sinusoidal dilation in rats [20,21,22,23,24,25]. MCT leads to histological changes in rat livers that are similar to those seen in patients with SOS, yet the adequate adaption of findings based on the MCT model to human patients remains unclear. Robinson et al. established a protocol using 5-fluorouracil, leucovorin, and oxaliplatin (FOLFOX), a common regimen regularly administered to patients with CLM [26], to induce SOS in mice [27,28]. FOLFOX-induced SOS might be more suitable for gaining insight into the pathogenesis, although conclusions about effects of oxaliplatin on the organism can only be made with certainty from a model that uses solely oxaliplatin. This is aggravated by the fact that 5-fluorouracil impacts the liver on its own [4,29]. To our knowledge, we present the first murine model of SOS induction by oxaliplatin alone, which might be limited from a point of generalizability to clinical regimens but provides the option of an isolated view on oxaliplatin-SOS effects. Similar to Robinson’s model, oxaliplatin was administered on a weekly basis over a period of five weeks; however, we identified a higher dose of oxaliplatin (11 mg/kg) required for the development of SOS. Animals were fed with a phytoestrogen-free diet, since phytoestrogens may protect against liver injury [30,31,32,33,34]. SOS was found in all of Robinson’s mice, whereas our model did not reach 100% efficacy as it was developed in only 90% of wt mice treated with oxaliplatin, a limitation that also applied for the MCT rat model of SOS [25]. However, the risk for underestimation of SOS has been limited in our study, as evaluation of slides was performed blinded to treatment and control groups.

Concomitant anti-VEGF therapy facilitated a reduced incidence of SOS development compared to treatment with oxaliplatin alone, as shown in wt but not in CD39-null mice. The protective effect of bevacizumab against oxaliplatin-induced SOS in patients with CLM has been previously reported [13,35,36,37,38]. The results of our study confirm these earlier reports and provide preclinical evidence on this topic. However, Jafari et al. recently reported contrary results from a rat model of MCT-induced SOS. They found that adding recombinant rat anti-VEGF to MCT led to a higher incidence of SOS, whereas concomitant recombinant rat VEGF protected against SOS development [39]. The authors explain the mechanism of this opposite finding with increased MMP-9 levels after concomitant treatment with MCT and anti-VEGF. Still, the MCT model may not adequately represent the pathological processes that take place in human patients treated with oxaliplatin-containing regimens.

In line with animal models [28] and studies of patients with and SOS [11,12], elevated levels of VEGF mRNA were found in liver tissue and plasma of mice after treatment with oxaliplatin in our study, yet the highest plasma levels of VEGF were measured in the OxAV groups. In contrast to plasma levels, we found comparable mRNA expression of VEGF-A between Ox and OxAV groups. High blood concentrations of VEGF have also been reported in patients receiving bevacizumab [40,41]. The rise of VEGF concentration in these patients may be considered a surrogate marker for optimal anti-VEGF dosing [42,43], and is likely caused by inactivation of circulating VEGF and subsequently reduced clearance [41,44]. Moreover, common measuring methods may often not be able to distinguish between free circulating, thereby active, and antibody-bound, thereby inactive, VEGF without the previous removal of IgG [44]. Consistent to these findings, we measured significantly higher plasma levels of VEGF-A in AV than in Glu groups while gene expression was found to be equivalent.

To date, the role of VEGF in the development of SOS is unclear, hence, we tried to elucidate the involvement of the VEGF pathway by analyzing the receptors VEGF-R1 and VEGF-R2. VEGF-R2 was found to be significantly upregulated in wtOxAV compared to wtOx, and VEGF-R2 was also downregulated in all CD39-null mice when compared to their respective wt treatment groups. Therefore, VEGF-R2 may have a potential role in the protective effect of a VEGF-inhibition against SOS development considering that all mice in the cd39OxAV group developed SOS. These findings give rise to the question of the protective efficacy of a targeted inhibition of VEGF-R2 against SOS development. While VEGF-R2 antagonists such as *ramucirumab* are under clinical investigation for the therapy of CLM [45], their effect on SOS development has not been reported so far. Nakamura et al. reported that rats treated with sorafenib, an inhibitor of several kinases including VEGF-R2, were protected against SOS in an MCT model [46]. They attributed this effect to the suppression of MMP-9 that is normally induced via the JNK-pathway upon activation of VEGF-R2 by VEGF. Indeed, a rise in MMP-9 in rodents and patients with SOS has been frequently reported [11,28,47]. However, our model of SOS did not show this phenomenon, since plasma MMP-9 levels were equivalent in all wt mice in our study. Additionally, TIMP-1, an inhibitor of MMP-9, showed no significant differences between wt groups. Interestingly, cd39Ox and cd39OxAV groups had the lowest values of MMP-9 and the highest values of TIMP-1 among CD39-null mice. This constellation is expected with regards to the downregulation of VEGF-R2 in CD39-null mice, and hence the missing activation of the JNK-pathway during high concentrations of VEGF.

Similar to partial hepatectomies for patients with CLM, MH was performed on wt mice after five weeks of treatment. Liver damage was assessed by liver transaminases and was more advanced in mice treated with oxaliplatin-based chemotherapy, as seen in other models of SOS [28]. In addition, increased preoperative AST may serve as a surrogate factor for SOS development [6,8,29]. VEGF inhibition reduced liver damage after MH in our study. For patients with CLM, concomitant treatment with bevacizumab in addition to oxaliplatin-based chemotherapy may prevent SOS, protect against postoperative liver insufficiency, and thereby improve short-term outcomes after liver resection [38]. Hyperbilirubinemia was mostly experienced in mice treated with oxaliplatin alone, whereas additional anti-VEGF treatment was associated with decreased bilirubin levels. Increased plasma levels of bilirubin may be used to predict [29] and diagnose SOS [48].

In addition to liver damage, we assessed the regenerative capacity of the liver after MH over a time period of three days by using the weights of resected specimen and immunohistological proliferation markers. Liver regeneration as measured by liver mass, and Ki-67 was significantly reduced after treatment with oxaliplatin compared to controls, which may be linked to the development of SOS in these mice. Additionally, HGF, a major cytokine involved in liver regeneration [49], was significantly reduced in the plasma of oxaliplatin-treated mice in comparison to controls. However, concomitant VEGF-inhibition was not able to achieve a significantly improved liver regeneration in comparison to treatment with oxaliplatin alone, although animals of this group reached the regenerative capacity of control mice at most time points after MH. These findings indicate that additional inhibition of VEGF may have the potential to improve hepatic regeneration by protecting against oxaliplatin-associated SOS development. An adequate liver regeneration is of major importance for patients undergoing liver surgery for CLM. So far, reports on the impact of oxaliplatin-induced SOS on liver regeneration after hepatectomy are scarce. Similar to our results, Hubert et al. performed hepatectomy after treatment with oxaliplatin in rats and found a reduced BrdU incorporation for three days after surgery in comparison to controls. This finding was not improved by additional bevacizumab administration [50]. In this study, however, sinusoidal changes were not seen in oxaliplatin-treated rats, and a human VEGF-antibody was used.

Our model and concluded findings may also provide insights for patients who develop SOS after hematopoietic stem cell transplantation (HSCT). SOS may occur at any time after HSCT for around 14% of patients; however, it is most common during the first three weeks [51]. Typical clinical symptoms include painful hepatomegaly, jaundice, ascites, and weight gain, and diagnosis may be made via the Baltimore [52], modified Seattle [53], or European Society for Blood and Marrow Transplantation criteria [54]. In addition, imaging techniques or liver biopsy may be needed to verify the diagnosis. Pathological changes in liver tissue are similar to those seen in patients with oxaliplatin-induced SOS: sinusoidal dilation with digestion by erythrocytes, hepatocellular necrosis, and fibrosis in late stages [55].

Our study has several limitations. Most importantly, our protocol was strongly limited by the general performance of the animals being negatively affected by the systemic treatment. Particularly, in the cd39Ox group, the additional stress due to the hepatectomy caused an unacceptable mortality rate after surgery in preliminary experiments. We attribute the high failure rate in CD39-null mice to the a priori impaired hepatic regenerative capacity [18]. Considering chemotherapy, liver injury due to SOS, MH, and, in case of CD39-null mice, preexisting conditions, we strongly believe that we operated at the limits of what is possible in a small animal model. However, with 11 mg/kg oxaliplatin, we identified the most appropriate dose for achieving a reasonable incidence of SOS while ensuring animal safety after surgery for wt mice. If animals were sacrificed instead of subjected to MH, more frequent and more advanced SOS would have certainly been possible with 12–14 mg/kg oxaliplatin over five weeks, as seen in our preliminary experiments. In addition, we decided to use a murine VEGF antibody instead of the human variant bevacizumab in order to guarantee an adequate neutralization of murine VEGF-A. The use of bevacizumab in mice for research purposes apart from xenograft models is still under debate [56,57]. Furthermore, a tumor model of CLM was not implemented in our study, though tumor-related interactions may play a role within the development of SOS [27]. In that way, our model misses potential contributions of colorectal cancer and CLM to SOS development; however, we aimed to establish a preclinical model of SOS induced by oxaliplatin alone without possible confounding factors. Lastly, results collected after MH may have been influenced by a potential learning curve on part of the surgeons. By alternating and randomizing treatment groups, we tried to challenge this issue. Nevertheless, a negative impact, especially on liver damage parameters, may still have been possible.

From a clinical perspective, our observations invoke complex questions to which extent bevacizumab should be used in the preoperative systemic therapy of CLM. While a protective effect on SOS might be beneficial, it has to be balanced against known side effects of bevacizumab such as bleeding and thrombosis, which may counteract the advantages of liver tissue protection. Furthermore, in the specific context of “conversion” therapy—aiming to make initially unresectable CLM resectable—regimens with high objective response rates are the preferred options, resulting in the use of triplet chemotherapy including irinotecan (FOLFOXIRI) or doublet regimens (FOLFOX/FOLFIRI) plus anti-EGFR antibodies when possible [58,59,60,61]. Unlike these drugs, the efficacy of bevacizumab in terms of objective response rates is under debate and may not provide higher conversion rates.

## 5. Conclusions

In this study, we have established a novel oxaliplatin-induced preclinical model of SOS in mice and showed the protective effect of a VEGF-inhibition against the development of SOS. In our model, this protective effect may be promoted by an upregulation of the receptor VEGF-R2. After hepatectomy, liver damage was decreased in mice who received an additional VEGF inhibition in comparison to mice treated with oxaliplatin alone. However, VEGF inhibition was not able to facilitate improved liver regeneration after chemotherapy. By targeting the VEGF pathway, the prohibition of SOS may be able to reduce postoperative morbidity in patients with CLM subjected for hepatectomy. Our findings require supporting evidence from human case series and correlation with other regimens including fluoropyrimidines, irinotecan and anti-EGFR antibodies.

## Figures and Tables

**Table 1:** Histopathological analysis of liver tissue of wt and CD39-null mice after five weeks of treatment, according to Rubbia-Brandt et al. [2].

Parameters	wtOx (*n* = 40)	wtOxAV (*n* = 40)	wtAV (*n* = 12)	wtGlu (*n* = 24)	*p*	cd39Ox (*n* = 24)	cd39OxAV (*n* = 12)	cd39AV (*n* = 12)	cd39Glu (*n* = 22)	*p*
Sinusoidal dilation, *n* (%)					<0.0001					<0.0001
absent	4 (10)	17 (42)	12 (100)	24 (100)		0 (0)	0 (0)	12 (100)	22 (100)	
present	36 (90)	23 (58)	0 (0)	0 (0)		24 (100)	12 (100)	0 (0)	0 (0)	
Grading of sinusoidal dilation, *n* (%)					<0.0001					<0.0001
Grade 0	5 (12)	18 (45)	12 (100)	24 (100)		0 (0)	0 (0)	12 (100)	22 (100)	
Grade 1	32 (80)	21 (53)	0 (0)	0 (0)		16 (66)	10 (83)	0 (0)	0 (0)	
Grade 2	3 (8)	1 (2)	0 (0)	0 (0)		4 (17)	2 (17)	0 (0)	0 (0)	
Grade 3	0 (0)	0 (0)	0 (0)	0 (0)		4 (17)	0 (0)	0 (0)	0 (0)	
Perisinusoidal hemorrhage, *n* (%)					0.019					0.233
absent	35 (88)	40 (100)	12 (100)	24 (100)		22 (92)	12 (100)	10 (83)	17 (77)	
present	5 (12)	0 (0)	0 (0)	0 (0)		2 (8)	0 (0)	2 (17)	5 (23)	
Nodularity, *n* (%)					-					-
Grade 0	0 (0)	0 (0)	0 (0)	0 (0)		24 (100)	12 (100)	12 (100)	22 (100)	
Grade 1	40 (100)	40 (100)	12 (100)	24 (100)		0 (0)	0 (0)	0 (0)	0 (0)	
Grade 2	0 (0)	0 (0)	0 (0)	0 (0)		0 (0)	0 (0)	0 (0)	0 (0)	
Grade 3	0 (0)	0 (0)	0 (0)	0 (0)		0 (0)	0 (0)	0 (0)	0 (0)	
Steatosis, *n* (%)					<0.0001					0.782
absent	24 (60)	32 (80)	12 (100)	24 (100)		23 (83)	12 (100)	12 (100)	21 (96)	
present	16 (40)	8 (20)	0 (0)	0 (0)		1 (4)	0 (0)	0 (0)	1 (4)	
Grading of steatosis, *n* (%)					0.045					0.529
Grade 0	28 (70)	33 (82)	12 (100)	24 (100)		24 (100)	12 (100)	12 (100)	21 (96)	
Grade 1	6 (15)	4 (10)	0 (0)	0 (0)		0 (0)	0 (0)	0 (0)	1 (4)	
Grade 2	2 (5)	3 (8)	0 (0)	0 (0)		0 (0)	0 (0)	0 (0)	0 (0)	
Grade 3	4 (10)	0 (0)	0 (0)	0 (0)		0 (0)	0 (0)	0 (0)	0 (0)	
Steatohepatitis, *n* (%)					-					-
absent	40 (100)	40 (100)	12 (100)	24 (100)		24 (100)	12 (100)	12 (100)	22 (100)	
present	0 (0)	0 (0)	0 (0)	0 (0)		0 (0)	0 (0)	0 (0)	0 (0)	
Hepatocellular damage, *n* (%)					0.001					0.062
absent	25 (63)	33 (82)	12 (100)	24 (100)		12 (50)	3 (25)	6 (50)	16 (73)	
present	15 (37)	7 (18)	0 (0)	0 (0)		12 (50)	9 (75)	6 (50)	6 (27)	

## Data Availability

The data presented in this study are available on request from the corresponding author.

## Appendix A. Administration of Chemotherapy, Major Partial Hepatectomy and Postoperative Care

Mice were held according to standard animal house care with ad libitum access to water in a 12-h light cycle and received a phytoestrogen-free diet (C 1077, Altromin, Lage, Germany) [28]. Oxaliplatin and anti-VEGF were given in weekly doses of 11 mg/kg and 10 mg/kg, respectively, as identified in preliminary experiments. Animals receiving oxaliplatin and anti-VEGF were injected on consecutive days. Control animals were administered 5% glucose that was given in a volume matching to oxaliplatin. All injections were performed in short-time inhalation anesthesia by isoflurane (AbbVie, Wiesbaden, Germany). Oxaliplatin (Medac, Wedel, Germany) was acquired from our institution’s pharmacy and diluted in 5% glucose (B. Braun, Melsungen, Germany) to obtain a solution of 1.1 mg/mL. The murine VEGF-A antibody was externally acquired (2G11-2A05, Biolegend, San Diego, CA, USA).

Mice receiving oxaliplatin were either sacrificed or subjected to MH (with *n* = 12 in each group) based on their general health after completion of treatment. Wild-type mice receiving glucose were randomly selected to either undergo MH or sham operation (*n* = 12 each). MH was performed as described by Higgins et al. adapted to the mouse [62]: After establishment of general anesthesia by 3.5% isoflurane and subcutaneous injection of 10 mg/kg ketamine (CP-Pharma, Burgdorf, Germany), 200 mg/kg metamizole (Sanofi-Aventis, Frankfurt, Germany) and 5 mg/kg carprofen (Zoetis, Berlin, Germany), median laparotomy was performed and the falciform ligament was partially dissected to mobilize the liver. Subsequently, the median and left lateral lobe of the liver were ligated and removed to achieve a hepatectomy of approximately 70%. The procedure was completed by separately suturing fascia and skin. Sham operations consisted of opening of the abdomen, dissecting of the falciform ligament without removing any tissue and carefully mobilizing the liver. Attention was paid to require approximately the same amount of time as MH. After surgery, mice were placed in a heating chamber (36 °C) until fully awoken and were then transferred in their pre-heated cage. Postoperative care consisted of visitations at regular intervals with assessment of body weight, signs of pain, and general performance. Daily subcutaneous injections of 5 mg/kg carprofen for analgesia were performed. Additionally, metamizole was added to the drinking water to obtain a solution of 7 mg/mL.

Mice subjected to MH were culled at different and randomly selected time points after surgery (24, 36, 48 or 72 h with *n* = 3 mice each). Two hours before sacrifice, 50 mg/kg bromodeoxyuridine (BrdU, Sigma-Aldrich, St. Louis, MO, USA) was injected intraperitoneally. Sacrifice was performed by establishing general anesthesia, opening of the abdomen, and removal of blood via heart puncture through the diaphragm into heparin-coated syringes (20 I.E. heparine/mL blood, Ratiopharm, Ulm, Germany). Blood was centrifuged (3000 rpm for 10 min) to obtain plasma, which was then frozen in liquid nitrogen.

## Appendix B. Processing of Specimen Including Histology, Liver Regeneration, Quantitative PCR, and Plasma Analysis

Histology. Liver specimen obtained during MH or sacrifice were weighed and then fixed in 4% formaldehyde (VWR, Radnor, PA, USA) or frozen in liquid nitrogen. Specimen fixed in formaldehyde were embedded in paraffin, sliced in 2 µm slices, and then stained with Mayer’s hematoxylin (AppliChem, Darmstadt, Germany) and eosin (Morphisto, Frankfurt, Germany; H&E).

Liver regeneration. Liver regeneration was approximated by estimating the liver weight increase from MH to sacrifice by calculating the relative liver mass increase, or relative hepatic regrowth, by assuming an MH of 70% (weight of median and left lateral lobe) and extrapolating this weight to 100%, relative to the body weight. The liver weight gain of the right and caudate lobe at sacrifice was expressed relatively to the extrapolated weight of the whole liver. Liver regeneration was also assessed by immunohistological staining for Ki-67 and BrdU. Sections were deparaffinized, rehydrated and subjected to a heat-induced antigen-demasking process with a 0.01 M citrate buffer (pH 6) followed by protein blocking (Protein Block Serum-Free, DAKO, Carpinteria, CA, USA) in case of BrdU staining. Thereafter, endogenous peroxidase activity was blocked by incubating the sections with a peroxidase blocking solution (DAKO, Carpinteria, CA, USA). Incubation with the primary antibody was done overnight (Ki-67: ab16667, Abcam, Cambridge, UK; BrdU: ab326, Abcam, Cambridge, UK). The next day, sections were incubated with a biotinylated secondary antibody (Biotinylated Link, DAKO, Carpinteria, CA, USA) followed by incubation with horseradish-conjugated streptavidin (DAKO, Carpinteria, CA, USA). Finally, sections were stained by the peroxidase substrate, 3,30-diaminobenzadine (DAKO, Carpinteria, CA, USA) and then dipped in Mayer’s hematoxylin for nuclear staining. Staining for Ki-67 and BrdU was quantified by using Fiji software [63] (an expanded version of ImageJ [64], version 2.0.0) using five pictures per slide in 40x magnification, and the ratio of stained to unstained nuclei was calculated (Ki-67 and BrdU indices).

Quantitative PCR. The RNeasy system (Qjagen, West Sussex, UK) was used according to the manufacturer’s instructions to isolate RNA from resected liver tissue. Two µg of RNA, reverse transcriptase, and random primers (Thermo Fisher Scientific, Waltham, MA, USA) were used to generate cDNA. Real-time PCR for VEGF-A, VEGF-R1, and VEGF-R2 was performed using TaqMan Gene Expression Master Mix (Thermo Fisher Scientific, Waltham, MA, USA) and a StepOne Plus cycler (Applied Biosystems, Waltham, MA, USA). Peptidylprolyl Isomerase A (PPIA) and β-Actin served as internal control. Gene expression was calculated using REST 2009 software (Qjagen, West Sussex, UK) [65]. Results were reported as fold change with 95% confidence intervals.

Plasma analysis. Plasma was analyzed for aspartate transaminase (AST), alanine transaminase (ALT), bilirubin, and albumin by our institution’s laboratory (Labor Berlin–Charité Vivantes GmbH). Bilirubin ≥0.15 mg/dL was defined as hyperbilirubinemia. Additional plasma testing for vascular endothelial growth factor (VEGF), hepatocyte growth factor (HGF), matrix metalloproteinase-9 (MMP-9), and tissue inhibitor of metalloproteinases-1 (TIMP-1) was performed using a magnetic bead-based multiplex assay according to the manufacturer’s protocol (Magpix, Luminex Corporation, Austin, TX, USA; premixed multi-analyte kit by R&D, Minneapolis, MN, USA).

## Appendix C. Detailed Results of Quantitative PCR Analysis

Comparing all wt groups, the expressions of VEGF-A (*p* < 0.0001) and its receptors VEGF-R1 (*p* = 0.025) and VEGF-R2 (*p* = 0.003) were significantly different. Normalized to wtGlu, expressions of VEGF-A, VEGF-R1, and VEGF-R2 were 1.6-fold (*p* = 0.002, 95% CI [0.4–10.3]), 0.8-fold (*p* = 0.172, 95% CI [0.2–4.7]), and 1.0-fold (*p* = 0.873, 95% CI [0.3–4.3]) for wtOx, 1.7-fold (*p* < 0.0001, 95% CI [0.4–8.0]), 1.0-fold (*p* = 0.931, 95% CI [0.2–5.4]), and 1.3-fold (*p* = 0.066, 95% CI [0.3–5.8]) for wtOxAV, and 1.1-fold (*p* = 0.477, 95% CI [0.3–5.0]), 0.8-fold (*p* = 0.111, 95% CI [0.3–3.5]), and 0.9-fold (*p* = 0.649, 95% CI [0.3–3.8]) for wtAV, respectively. Post-hoc analysis revealed the up-regulation of VEGF-A in wtOx (*p* = 0.004) and wtOxAV mice (*p* < 0.0001) compared to controls. Between wtOx and wtOxAV, expressions of VEGF-A (*p* = 0.786) and VEGF-R1 (*p* = 0.409) were comparable. However, VEGF-R2 was upregulated in wtOxAV compared to controls (*p* = 0.045). In addition, VEGF-R1 (*p* = 0.042) and -R2 (*p* = 0.002) were higher expressed in wtOxAV than in wtAV.

In the comparison of all CD39-null groups, results were significantly different for VEGF-A (*p* < 0.0001) and VEGF-R1 (*p* < 0.0001), but not for VEGF-R2 (*p* = 0.112; Figure 4). Fold-change calculation of VEGF-A and VEGF-R1 normalized to the results of cd39Glu showed 1.8-fold change (*p* < 0.0001, 95% CI [0.6–4.7]) and 1.2-fold change (*p* = 0.137, 95% CI [0.4–2.9]) for cd39Ox, 1.9-fold change (*p* < 0.0001, 95% CI [0.9–4.0]) and 1.8-fold change (*p* < 0.0001, 95% CI [0.9–3.7]) for cd39OxAV, and 0.9-fold change (*p* = 0.291, 95% CI [0.5–2.0]) and 0.8-fold change (*p* = 0.007, 95% CI [0.3–1.3]) for cd39AV, respectively. Similar to wild-type mice, the post-hoc analysis showed the upregulation of VEGF-A in cd39Ox (*p* < 0.0001) and cd39OxAV (*p* < 0.0001), whereas no significant differences were found between these two groups (*p* = 0.990). Interestingly, VEGF-R1 was upregulated in cd39OxAV compared to cd39Ox (*p* = 0.042) and cd39AV (*p* < 0.0001).

In the final comparison of wild-type vs. CD39-null groups, VEGF-A (*p* < 0.0001), VEGF-R1 (*p* < 0.0001) and VEGF-R2 (*p* < 0.0001) were all significantly different expressed. Comparing each respective treatment group with each another, VEGF-A mRNA was equivalently expressed in Ox (*p* = 0.466), OxAV (*p* = 0.570) and Glu (*p* = 0.113) groups but downregulated in cd39AV (0.7-fold change, *p* = 0.005, 95% CI [0.2–1.7]). VEGF-R1 (0.7-fold change, *p* = 0.042, 95% CI [0.1–3.3]) and VEGF-R2 (0.6-fold change, *p* < 0.0001, 95% CI [0.2–2.1]) were downregulated in cd39Ox in comparison to wtOx. Downregulation of VEGF-R1 was detected in all CD39-null control groups as well (cd39AV: 0.5-fold change, *p* < 0.0001, 95% CI [0.2–1.0]; cd39Glu: 0.5-fold change, *p* < 0.0001, 95% CI [0.2–1.9]), but not in cd39OxAV (*p* = 0.488). VEGF-R2 was also downregulated in all other CD39-null groups (cd39OxAV: 0.5-fold change, *p* < 0.0001, 95% CI [0.2–1.5]; cd39AV: 0.6-fold change, *p* = 0.001, 95% CI [0.2–1.3]; cd39Glu: 0.6-fold change, *p* < 0.0001, 95% CI [0.2–1.9]).

For detailed cycle threshold values please refer to Table A1.

**Table A1:** Overview of cycle threshold values of quantitative PCR analysis.

Groups	Cycle Threshold Values		
	VEGF-A	VEGF-R1	VEGF-R2
**wtOx**	23.41, 22.58, 22.47, 23.14, 22.52, 22.98, 23.12, 23.62, 22.09, 23.15, 22.89, 22.81, 22.83, 22.72, 21.48, 22.84, 22.91, 22.93, 22.65, 23.03, 22.32, 23.26, 22.59, 22.64, 23.64, 22.46, 21.3, 22.38, 22.33, 22.66, 23.43, 22.9, 22.25, 22.91, 22.44, 22.33, 22.46, 22.41, 22.84, 21.89	28.7, 27.05, 26.75, 27.21, 27.18, 27.97, 27.47, 29.01, 27.84, 27.88, 27.62, 27.18, 27.14, 28.32, 26.63, 27.21, 26.69, 26.64, 26.41, 26.97, 26.49, 27.09, 26.97, 26.39, 28.17, 26.69, 26.6, 26.51, 25.87, 27.29, 27.54, 27.28, 26.45, 27.57, 27.1, 27.36, 28.03, 26.36, 27.23, 26.06	25.68, 24.54, 24.69, 24.88, 24.73, 24.65, 25.59, 26.2, 24.31, 24.96, 24.89, 24.95, 24.71, 25.21, 24.65, 24.85, 24.55, 24.94, 24.3, 24.84, 24.83, 25.06, 25.23, 24.42, 26.02, 24.9, 26.45, 24.44, 23.92, 24.73, 25.17, 24.57, 24.13, 24.83, 24.83, 25, 25.09, 24.18, 24.86, 24.01
**wtOxAV**	23.03, 24.83, 24.42, 22.74, 22.08, 22.2, 23.27, 23.79, 23.39, 23.37, 23.68, 23.17, 23.61, 22.8, 23.7, 22.27, 22.72, 23.58, 22.59, 23.07, 22.69, 23.37, 22.73, 23.1, 22.5, 22.94, 23.43, 21.89, 22.47, 22.69, 22.78, 21.72, 22.84, 22.96, 22.78, 22.85, 22.76, 22.66	28.24, 29.08, 30.11, 27.95, 26.19, 27.63, 27.34, 28.71, 27.12, 27.14, 27.61, 27.74, 27.83, 26.81, 26.96, 24.78, 26.04, 27.03, 26.94, 27.51, 28.15, 27.55, 27.28, 27.98, 26.67, 27.35, 28.45, 26.97, 27.17, 27.4, 26.96, 25.29, 27.83, 27.73, 27.35, 27.58, 26.94, 26.94	25.52, 26.62, 27.02, 24.6, 24.41, 25.11, 24.69, 26.03, 24.86, 25.42, 25.14, 25.01, 24.98, 24.32, 24.87, 24.76, 23.71, 24.51, 23.68, 25, 24.69, 25.01, 24.93, 25.11, 24.21, 25.16, 25.88, 24.34, 24.53, 24.41, 24.29, 23.54, 24.87, 24.8, 24.38, 24.98, 24.05, 23.95
**wtAV**	23.9, 23.41, 24.2, 23.64, 22.77, 23.49, 23.44, 23.85, 23.92, 23.67, 23.55, 23.73	26.79, 27.77, 27.39, 27.35, 27.45, 27.49, 27.28, 27.9, 28.26, 27.4, 27.01, 27.86	24.61, 25.45, 25.39, 25.15, 24.41, 25.69, 25, 25.29, 25.47, 25.25, 25.12, 25.42
**wtGlu**	23.78, 24.35, 24.73, 23.61, 23.82, 23.23, 23.79, 23.56, 23.3, 23.33, 24.38, 23.16	27.47, 28, 28.07, 27.74, 28.04, 26.69, 26.85, 26.8, 26.01, 26.92, 27.72, 26.37	24.59, 25.83, 25.79, 25.33, 24.76, 24.92, 24.91, 24.56, 24.66, 24.71, 25.56, 24.27
**cd39Ox**	22.15, 22.06, 22.95, 22.99, 22.91, 23.49, 22.85, 22.36, 22.48, 23.96, 22.68, 22.61, 21.96, 21.73, 22.11, 22.03, 21.79, 22.33, 21.44, 21.97, 23.29, 21.07, 22.33, 21.34	26.15, 26.73, 27.58, 27.76, 29.48, 28.74, 26.96, 26.35, 26.31, 27.91, 26.64, 27.45, 26.65, 26.64, 26.99, 27.33, 26.59, 27.41, 26.18, 26.81, 28.3, 27.02, 28.07, 26.39	24.57, 24.9, 25.05, 25.07, 26.99, 25.54, 24.96, 24.6, 24.33, 24.92, 24.27, 24.87, 23.98, 24.09, 24.68, 24.57, 24.48, 24.62, 24.2, 25.93, 26.03, 25.88, 25.36, 25.27
**cd39OxAV**	21.25, 20.53, 21.83, 21.05, 20.98, 21.56, 22.73, 21.94, 22.02, 22.28, 22.95, 21.49	25.47, 24.86, 25.85, 25.53, 25.26, 25.92, 26.78, 26.34, 26.24, 26.55, 28.26, 26.44	23.96, 23.32, 23.95, 23.45, 23.75, 24.4, 25.22, 24.9, 24.13, 25.07, 26.04, 24.56
**cd39AV**	22.27, 23.77, 22.92, 23.43, 22.37, 22.88, 22.82, 23.21, 24.67, 21.87, 22.99, 23.51	26.34, 27.64, 27.45, 27.56, 26.35, 26.71, 26.55, 27.95, 29.82, 26.64, 27.94, 28.84	23.92, 25.56, 24.96, 24.69, 23.61, 24.02, 23.94, 25.07, 27.38, 23.92, 25.14, 26.22
**cd39Glu**	23, 23.03, 23.37, 23.99, 23.78, 24.01, 23.68, 22.75, 22.91, 23.25, 23.44, 23.32, 22.81, 24, 22.47, 22.98, 21.99, 22.15, 22.77, 22.27, 22.94, 22.94	26.91, 26.9, 27.46, 28.03, 28.24, 27.83, 27.64, 26.76, 26.98, 27.83, 27.91, 27.79, 27.41, 27.87, 26.74, 27.23, 26.41, 27.25, 27.44, 26.88, 27.14, 27.34	24.33, 24.44, 25.38, 25.94, 26.61, 25.36, 25.35, 24.22, 24.49, 25.43, 25.21, 24.82, 24.5, 25.31, 23.91, 24.89, 23.95, 24.95, 25.17, 24.22, 24.89, 25.02

## Appendix D. Detailed Results of Magnetic Bead-Based Blood Plasma Analysis

Analysis of wt mice at time of sacrifice revealed significant differences for VEGF-A (*p* < 0.0001) and HGF (*p* = 0.030), and comparable results for MMP-9 (*p* = 0.194) and TIMP-1 (*p* = 0.155). Median VEGF-A was highest for wtOxAV that was significantly increased in comparison to wtOx (*p* = 0.013) and wtGlu (*p* < 0.0001), but not to wtAV (*p* = 0.122). Control wt mice that received anti-VEGF alone showed increased values of VEGF-A than glucose receiving control mice (*p* = 0.020). HGF was significantly reduced in wtOx compared to wtGlu (*p* = 0.017).

Among CD39-null mice, VEGF-A (*p* < 0.0001), HGF (*p* < 0.0001), MMP-9 (*p* < 0.0001), and TIMP-1 (*p* < 0.0001) were found to be significantly different. Median VEGF-A was significantly increased in cd39OxAV (vs. cd39Ox, *p* = 0.011, and vs. cd39Glu, *p* = 0.002); however, it was equivalent to cd39AV (*p* = 0.193). The difference of VEGF-A between cd39Ox and cd39Glu just failed to reach statistical significance (*p* = 0.054). HGF was significantly reduced in cd39Ox mice (vs. cd39AV, *p* = 0.001, and vs. cd39Glu, *p* < 0.0001). MMP-9 was significantly reduced in cd39Ox (vs. cd39AV: *p* = 0.048) and cd39OxAV (vs. cd39AV: *p* = 0.003; vs. cd39Glu: *p* = 0.005). Contrarily, TIMP-1 was significantly increased in cd39Ox (vs. cd39AV: *p* = 0.010; vs. cd39Glu: *p* = 0.025). However, HGF (*p* = 1), MMP-9 (*p* = 1), and TIMP-1 (*p* = 1) were comparable between cd39Ox and cd39OxAV.
